# Breathing Monitoring in Soccer: Part I—Validity of Commercial Wearable Sensors

**DOI:** 10.3390/s24144571

**Published:** 2024-07-15

**Authors:** Lorenzo Innocenti, Chiara Romano, Giuseppe Greco, Stefano Nuccio, Alessio Bellini, Federico Mari, Sergio Silvestri, Emiliano Schena, Massimo Sacchetti, Carlo Massaroni, Andrea Nicolò

**Affiliations:** 1 Department of Movement, Human and Health Sciences, University of Rome “Foro Italico”, 00135 Rome, Italy; lorenzo.uniroma4@gmail.com (L.I.); g.greco1@studenti.uniroma4.it (G.G.); stefano.nuccio@uniroma4.it (S.N.); alessiobellini1@gmail.com (A.B.); federico.mari@uniroma4.it (F.M.); massimo.sacchetti@uniroma4.it (M.S.); andrea.nicolo@uniroma4.it (A.N.); 2 Department of Engineering, Unit of Measurements and Biomedical Instrumentation, Università Campus Bio-Medico di Roma, Via Alvaro del Portillo, 21, 00128 Rome, Italys.silvestri@unicampus.it (S.S.); e.schena@unicampus.it (E.S.); 3 Fondazione Policlinico Universitario Campus Bio-Medico, Via Alvaro del Portillo, 200, 00128 Rome, Italy

**Keywords:** wearables, exercise, respiratory rate, validity, algorithms, sport, breathing frequency, intermittent exercise, effort

## Abstract

Growing evidence suggests that respiratory frequency (*f_R_*) is a valid marker of effort during high-intensity exercise, including sports of an intermittent nature, like soccer. However, very few attempts have been made so far to monitor *f_R_* in soccer with unobtrusive devices. This study assessed the validity of three strain-based commercial wearable devices measuring *f_R_* during soccer-specific movements. On two separate visits to the soccer pitch, 15 players performed a 30 min validation protocol wearing either a ComfTech^®^ (CT) vest or a Bioharness^TM^ (BH) 3.0 strap and a Tyme Wear^TM^ (TW) vest. *f_R_* was extracted from the respiratory waveform of the three commercial devices with custom-made algorithms and compared with that recorded with a reference face mask. The *f_R_* time course of the commercial devices generally resembled that of the reference system. The mean absolute percentage error was, on average, 7.03% for CT, 8.65% for TW, and 14.60% for BH for the breath-by-breath comparison and 1.85% for CT, 3.27% for TW, and 7.30% for BH when comparison with the reference system was made in 30 s windows. Despite the challenging measurement scenario, our findings show that some of the currently available wearable sensors are indeed suitable to unobtrusively measure *f_R_* in soccer.

## 1. Introduction

The growing availability of wearable sensors measuring physiological and mechanical variables is reshaping the way athletes are monitored during training and competitions. This is of great importance for maximizing performance, optimizing training, and minimizing the risk of injury [1,2,3]. Soccer is benefiting substantially from the support of technology, because the stochastic nature of the game makes it otherwise challenging to describe the demand imposed by exercise on soccer players. For instance, the use of Global Positioning Systems (GPS) is widespread at elite and sub-elite levels [4,5]. Hence, we can acquire detailed information on the players’ movements in terms of distance covered, speed, accelerations, decelerations, changes of direction, and related information [4,5,6,7,8,9]. However, the individual response to exercise is best captured by physiological variables describing how the athlete reacts to a given external load [10,11,12,13]. Indeed, the physiological responses of soccer players performing the same activity might differ largely because of differences in fitness levels [14,15], among other factors. However, physiological monitoring in soccer is losing momentum because heart rate (HR)—the main variable currently recorded—does not effectively quantify the physical effort of a soccer player [16,17,18,19].

Emerging evidence suggests that respiratory frequency ($f_{R}$) monitoring may solve the problems observed when monitoring HR to a good extent. Indeed, $f_{R}$ shows a fast response to the alternation of work and rest phases during intermittent exercise, as opposed to the delayed response observed for HR both at the onset and offset of a short exercise bout [17,20,21,22]. Furthermore, unlike HR, $f_{R}$ reflects the physical effort of the players during the so-called supramaximal efforts [17], which are common events in soccer and other team sports. Moreover, $f_{R}$ is better associated with perceived exertion and exercise tolerance than HR, oxygen uptake, and blood lactate in different exercise modalities and conditions, including running-based activities [17,20,21,23]. The importance of $f_{R}$ monitoring deserves further consideration in light of the progressive development of wearable and unobtrusive technologies that can monitor breathing variables during exercise [23,24].

There is an abundance of contact-based methods that can be used to monitor $f_{R}$ [24,25,26,27,28,29,30,31,32]. Among these, the sensors measuring respiratory-induced torso movements are particularly suitable for breathing monitoring in soccer, as they can be integrated into straps or clothes used during training and competitions. For instance, soccer players commonly wear vests to allocate the GPS unit, and the vests can be integrated with strain sensors measuring $f_{R}$. Different commercial strain sensors have been tested during exercise, and some of them also during running activities [33,34,35,36,37,38,39]. For instance, the Bioharness^TM^ (BH) chest strap has been tested in different exercise studies [33,34,39]. However, we are unaware of studies that have specifically assessed the validity of wearable sensors measuring $f_{R}$ during soccer-specific activities. This is important because the signal-to-noise ratio of respiratory signals recorded with strain sensors may be largely affected by motion artifacts, which are sports-specific. Soccer is a particularly challenging scenario because it is characterized by unorthodox movements, changes of direction, and torso rotations that may impair the quality of the respiratory signal. It is also very important to assess the validity of breathing sensors on a breath-by-breath basis because soccer-based movements usually last a few seconds and $f_{R}$ may thus show large fluctuations in short time periods [17]. Hence, the commonly used approach for validating sensors of segmenting the signal into windows of several seconds (e.g., 30 s) is not sufficient in this scenario.

The purpose of this study was to assess the validity of three commercial devices measuring $f_{R}$ during soccer-specific movements. We designed a protocol including a warm-up with the ball and intermittent shuttle runs, and the validation was performed both on a breath-by-breath basis and using windows ranging from 1 s to 60 s. This was performed to investigate the effect of window length selection on the error of $f_{R}$ measurement. All of the commercial devices integrated strain sensors into either straps (i.e., BH) or vests (i.e., those from ComfTech s.r.l.^®^ and Tyme Wear^TM^ companies) and were thus chosen based on their potential suitability for soccer monitoring from a wearability perspective. We aimed to verify whether these devices were also effective in providing a valid measure of $f_{R}$, with important implications for the assessment and management of soccer players during training and competitions.

## 2. Materials and Methods

### 2.1. Experimental Set-Up and Protocol

This study tested the validity of three commercial wearable sensors measuring $f_{R}$ during soccer-specific movements. The commercial sensors were a BH strap (Medtronic, Boulder, CO, USA), a Tyme Wear^TM^ (TW) vest (Tyme Wear^TM^, Boston, MA, USA), and a ComfTech^®^ (CT) vest (Howdy Senior, ComfTech s.r.l.^®^, Monza, Italy). As the two wearable vests could not be used at the same time, participants performed the same validation protocol in two randomized visits to the soccer pitch. During one visit, participants were asked to wear the BH strap and the TW vest, while during the other visit they wore the CT vest. During both visits, the reference respiratory signal was registered with a previously validated custom-made wearable mask [22]. Fifteen male volunteers (mean ± standard deviation (SD): age 23 ± 4 years, height 176 ± 5 cm, body mass 69.1 ± 6.1 kg) participated in this study. They were healthy amateur soccer players with no recent injuries that may have impaired their participation. The study was approved by the Institutional Review Board of the University of Rome “Foro Italico” (CAR 149/2023) and conducted in accordance with the Declaration of Helsinki. All participants provided written informed consent.

The 30 min validation protocol was designed to evaluate the performance of the three commercial devices during soccer-specific movements. The synchronization between the respiratory signal from the commercial devices and the reference respiratory signal was guaranteed by performing previously described breathing maneuvers composed of three fast and deep breaths followed by 5 s of apnea [22]. These maneuvers were performed at the beginning, middle, and end of the validation protocol to ensure correct synchronization with the reference signal. The protocol was composed of four main phases.

1. *A paced-breathing test of 5 min*. This phase was included to systematically test the performance of the three commercial devices at different $f_{R}$ values. Participants were asked to run at a moderate self-paced speed while pacing their $f_{R}$ according to a metronome track beeping from 15 bpm to 75 bpm (the inspiratory and expiratory phases had two different tones to facilitate the execution of the task). Participants received the output of the digital metronome through in-ear headphones connected to a smartphone attached to their upper arm.
2. *A warm-up with the ball of 5 min*. This phase was included to test the performance of the three commercial devices during classical movements made by soccer players when passing the ball and running with it.
3. *A shuttle run intermittent test of 9 min*. This test reproduced the protocol performed in a previous study composed of 15 s of work and 30 s of passive recovery [17]. The test consisted of 12 shuttle runs performed according to a maximal session effort prescription. This test was chosen to verify whether the three commercial devices are suitable for describing the fast response of $f_{R}$ to the alternation of work and rest commonly observed during this test [17].
4. *A cool-down of 5 min*. This test phase was included to evaluate the performance of the three commercial devices during a slow recovery run.

### 2.2. Wearable Devices and Related Respiratory Signals

#### 2.2.1. Reference System

The reference system used is a custom-made wearable face mask integrating a thermistor collecting the temperature of the airflow exhaled by the nose and mouth [22]. This device showed excellent performance in measuring $f_{R}$ when compared to a flowmeter; mean absolute percentage error (MAPE) values lower than 3% were observed during intermittent exercise when the comparison was made on a breath-by-breath basis [22]. The respiratory waveform recorded with this device has a sampling frequency of about 50 Hz.

#### 2.2.2. ComfTech^®^ Vest

This vest integrates a resistive sensor recording respiratory-induced torso movements. An electronic unit detects and transmits the raw respiratory signal (sampled at about 13 Hz) to a mobile app through Bluetooth.

#### 2.2.3. Tyme Wear^TM^ Vest

This vest integrates a capacitive sensor recording respiratory-induced torso movements. A pod collects and streams data to a mobile app through Bluetooth, and a raw respiratory signal sampled at 25 Hz can be extracted.

#### 2.2.4. Bioharness^TM^ 3.0 Strap

This chest strap integrates a strain sensor recording respiratory-induced torso movements. An electronic module attached to the left side of the strap stores respiratory raw data sampled at 25 Hz.

### 2.3. Signal Pre-Processing

Data were processed with the MATLAB^®^ version 2023a (Mathworks, Inc., Natick, MA, USA). Signal pre-processing consisted of two main steps. First, the reference signal and the signals collected with the three wearable devices (i.e., CT, TW, and BH) were synchronized. For this purpose, all signals were cut from the breath preceding the apnea phase of the synchronization maneuver. Second, all synchronized signals were filtered with a first-order Butterworth band-pass filter with cutting frequencies of 0.01 Hz and 2 Hz, preserving the signal frequencies within the $f_{R}$ range usually observed during exercise.

### 2.4. Data Analysis and Respiratory Rate Estimation

**Figure 1:**
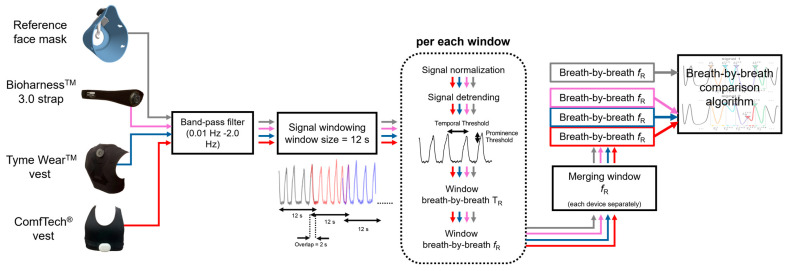
Flow chart of the pre-processing and analysis of the respiratory waveform leading to breath-by-breath comparison of *f_R_* between the reference system and the three commercial devices. T_R_, respiratory period.

After signal pre-processing, $f_{R}$ was extracted from the respiratory signals collected with the reference system and the wearable devices with algorithms working in the time domain (see Figure 1). Given the need to tailor algorithms to the specific demands of different sporting activities (soccer in this instance) and the peculiarities of each respiratory signal [39], the algorithm extracting $f_{R}$ was refined to maximize the performance of each wearable device, as detailed below.

**Figure 2:**
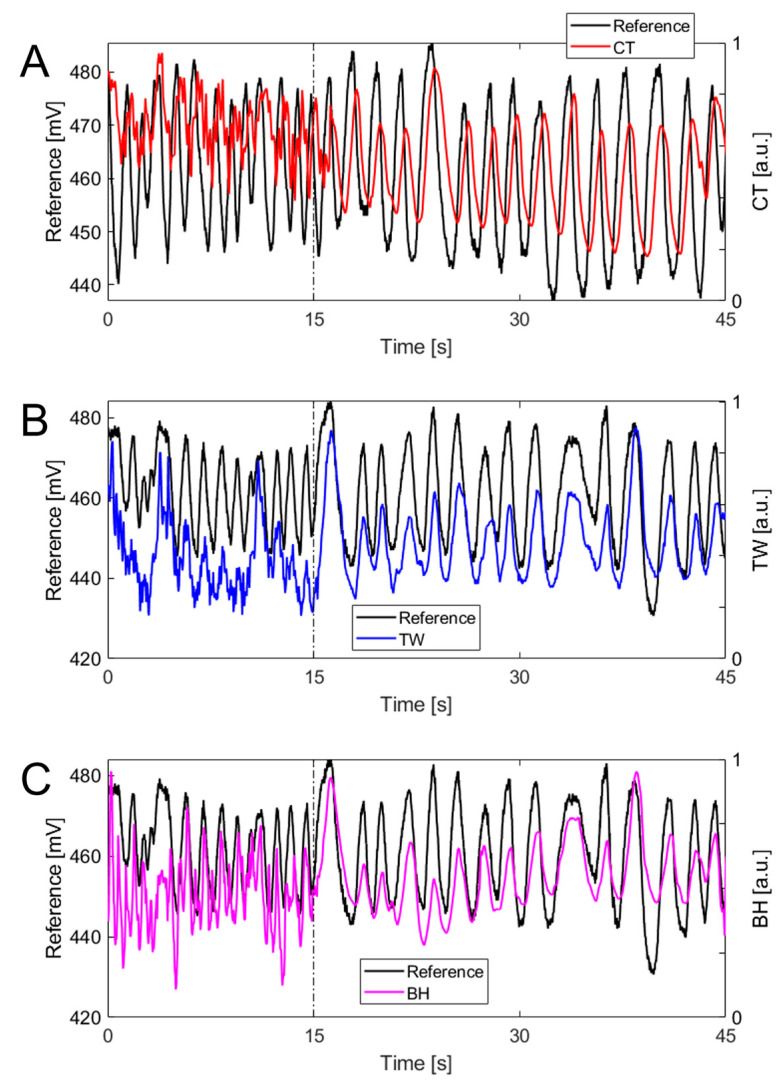
Comparison of the raw reference signal (in black) and the raw signal of the CT vest (**A**), the TW vest (**B**), and the BH strap (**C**) for a single participant. Data represent the first 45 s of the intermittent test. The vertical dashed and dotted line separates the 15 s of work from the 30 s of rest.

For the signals collected with the three commercial devices or the reference system, maximum peaks, corresponding to the end of the inspiratory phase, were identified and selected. The identification of each breath was made based on moving windows of 12 s with an overlap of 2 s. The window size allowed us to include at least two breaths (typically around 12 breaths/min in resting conditions), while the overlap was necessary to ensure that all breaths were considered in the analysis. In each window, signal normalization was performed, and the best straight-fit line was then removed from the data. Both of these steps were implemented to remove any signal drift not related to respiratory activity. Subsequently, an algorithm based on temporal and amplitude criteria was implemented to exclude artifacts, which may affect the respiratory waveform recorded with devices measuring the deformations of the rib cage. Indeed, the movement of the athlete may cause changes in the sensor output that are not related to the respiratory activity. However, some motion artifacts can be excluded by considering both physiological aspects (e.g., physiological values of respiratory rate) and morphological aspects of the signal (e.g., the amplitude of the waveform), as described below. The temporal criterion considered two consecutive peaks as separate events if the distance between them exceeded a minimum value set at 0.5 s. The amplitude criterion was individualized for each device because the morphology of the respiratory waveform changed substantially across devices (see Figure 2). For the reference system, we set a prominence threshold of 2% of the peak-to-peak maximum amplitude, in line with a previous study [22]. Only the peaks exceeding this threshold were selected as valid breaths, while the others were disregarded. For the three commercial devices, the amplitude criterion for peak identification was individualized for each device based on the assessment of the algorithm outcome to changes in prominence percentages set at 1%, 2%, 5%, 10%, 15%, and 20%. For each prominence percentage, false positives (i.e., breaths detected by the device but not by the reference system) and false negatives (breaths detected by the reference system but not by the device) were calculated and used to select a specific threshold for each device, as detailed below.

After peak detection, breath-by-breath $f_{R}$ was computed as the ratio between 60 and each respiratory period (i.e., the time interval between consecutive maximum peaks) for both the reference signal and the signals obtained from the commercial devices.

### 2.5. Breath-by-Breath Comparison

The breath-by-breath comparison between the reference system and each device under validation was performed using a previously presented algorithm [40]. This algorithm addresses mismatches in breath count detected between the reference system and the device being tested, an issue frequently observed in validation studies [40]. Notably, the algorithm identifies and excludes false positives and false negatives, hence including only true positives (breaths detected both by the device and the reference system) in the breath-by-breath comparison.

Specifically, let $N_{R}$ and $N_{D}$ be the number of breaths identified in the reference and device signals, respectively. For each breath extracted from the reference signal ($L_{R}^{i},i=1:N_{R}$), the time distances between it and the breaths identified in the device signal ($L_{D}^{j},j=1:N_{D}$) were computed. Then, the respiratory periods were computed for the reference as $T_{R}^{i}=L_{R}^{i+1}-L_{R}^{i}$ and for the device as $T_{D}^{j}=L_{D}^{j+1}-L_{D}^{j}$. Finally, to identify whether a reference breath was correctly captured by the device, the following conditions were checked.

1. True positives: for any $L_{R}^{i}$, the nearest $L_{D}^{j}$ was considered a true positive if it fell within ${[L}_{R}^{i}-\frac{T_{R}^{i}}{2},L_{R}^{i}+\frac{T_{R}^{i}}{2}]$. In such cases, the $f_{R}$ values were computed as the ratio between 60 and $T_{R}^{i}$ or $T_{D}^{j}$.
2. False positives: (i) the nearest $L_{D}^{j}$ of any $L_{R}^{i}$ was counted as a false positive if it did not fall within ${[L}_{R}^{i}-\frac{T_{R}^{i}}{2},L_{R}^{i}+\frac{T_{R}^{i}}{2}]$; (ii) any $L_{D}^{j}$ that was not the nearest of any reference $L_{R}^{i}$ was also counted as a false positive.
3. False negatives: finally, when $L_{R}^{i}$ did not have any nearest $L_{D}^{j}$ falling within ${[L}_{R}^{i}-\frac{T_{R}^{i}}{2},L_{R}^{i}+\frac{T_{R}^{i}}{2}]$, a false negative was counted.

### 2.6. Identification of the Prominence Percentage for Each Commercial Device

For each prominence percentage tested, the MAPE was computed from breath-by-breath values considering the entire validation protocol. In addition, false positives and false negatives were counted as detailed above and expressed as a percentage of the total breaths detected by the reference system. MAPE values were also calculated for each phase of the paced-breathing protocol, which was subdivided into ten portions, each representing 10% of total breaths. This protocol was chosen because a systematic increase in $f_{R}$ from 15 bpm to 75 bpm favors the understanding of how the prominence percentage affects MAPE values for different $f_{R}$ levels. The outcome of these analyses allowed us to identify a prominence threshold for each commercial device, as reported in the Results section. Once the prominence threshold was established for each commercial device, breath-by-breath comparison with the reference system was also performed by computing the Mean Absolute Error (MAE), the Mean of Differences (MOD), and the Limits of Agreements (LoAs), as performed in previous validation studies [22,41].

### 2.7. Comparison Based on Second-by-Second Values and Different Window Lengths

To compare the time course of $f_{R}$ measured with the reference system with that of $f_{R}$ measured with the commercial devices, breath-by-breath values were linearly interpolated and extrapolated every second. During the intermittent test, second-by-second $f_{R}$ data were plotted both as a function of time and as a function of the work–rest cycle (15 s of work and 30 s of rest), as previously detailed [17]. Second-by-second values were also averaged every 5 s, 10 s, 20 s, 30 s, and 60 s to compute MAPE values for each of the four phases composing the validation protocol.

## 3. Results

### 3.1. Identification of the Prominence Threshold for Each Commercial Device

**Figure 3:**
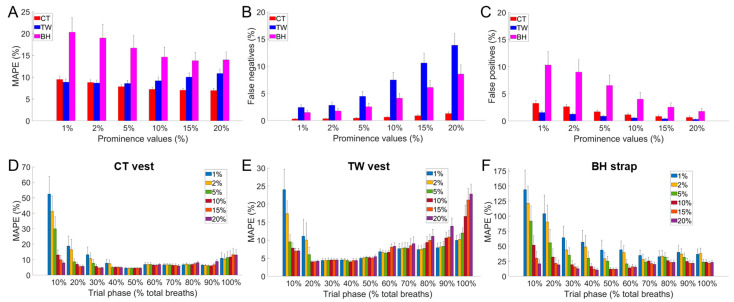
MAPE values (**A**), false negatives (**B**), and false positives (**C**) for different prominence percentage values for the CT vest (red bars), the TW vest (blue bars), and the BH strap (magenta bars). Breath-by-breath values of the entire validation protocol were included in the analysis. The lower panels show MAPE values for the different phases of the paced-breathing protocol (each phase contains 10% of total breaths) for the CT vest (**D**), the TW vest (**E**), and the BH strap (**F**). Data are mean ± SEM.

**Figure 4:**
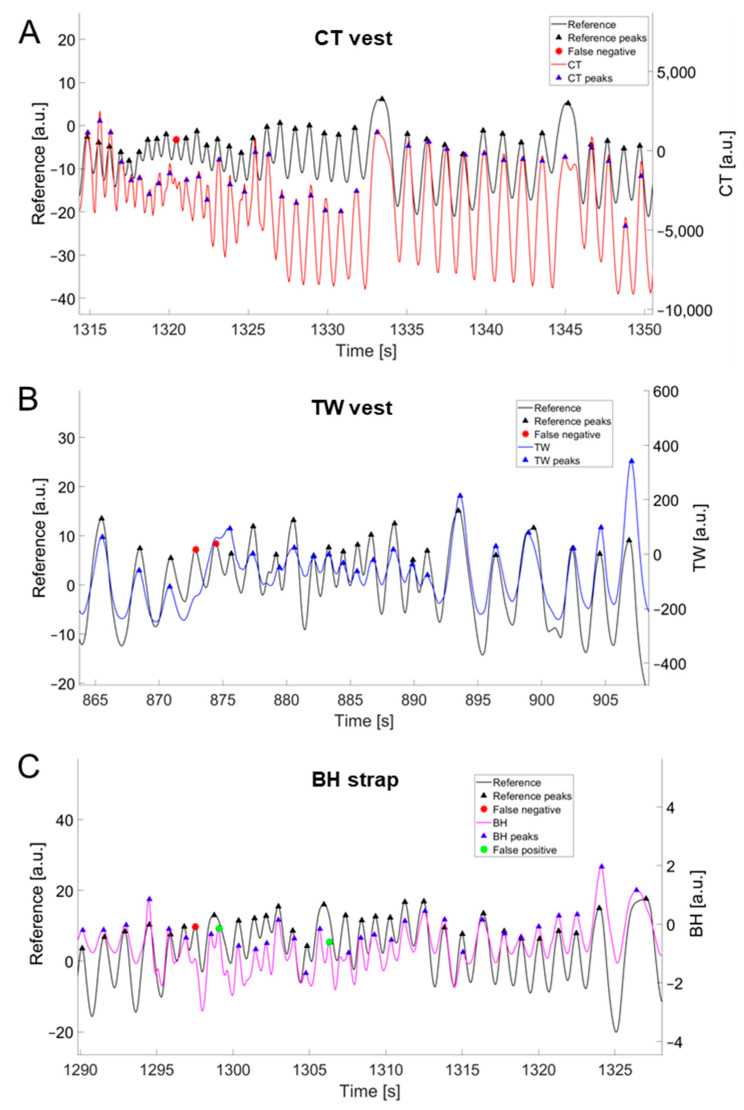
Examples of the comparison between the filtered reference signal (in black) and the filtered signals of the CT vest (**A**), the TW vest (**B**), and the BH strap (**C**). Peaks detected are marked with triangles, while false negatives and false positives are marked with red and green asterisks, respectively.

Changes in the prominence values (from 1% to 20%) affected the breath-by-breath MAPE values of the three commercial devices in different ways (Figure 3). The increase in prominence percentage resulted in a decrease in average MAPE values for BH and CT, while an increase in MAPE was observed for TW (Figure 3A). This is because the TW vest showed a substantial increase in false negatives with the increase in prominence percentage values, while false negatives were lower for BH and especially for CT. Figure 4 shows an example of the identification of false negatives and/or false positives for each of the three commercial devices. The Figure outlines how the identification of false positives or false negatives depends on the morphology of the respiratory waveform of the device under validation. Results from the paced-breathing phase suggest that *f*_R_ levels moderate the effect of changes in prominence percentage on MAPE. For instance, TW showed the highest MAPE values at 1% of prominence for low *f*_R_ values and at 20% of prominence for high *f*_R_ values. Hence, the choice of the prominence percentage for each device was based on a trade-off between false positives, false negatives, overall MAPE values, and MAPE values at different *f*_R_ levels. The prominence threshold identified was 10% for CT, 2% for TW, and 10% for BH.

### 3.2. Respiratory Frequency Time Course

**Figure 5:**
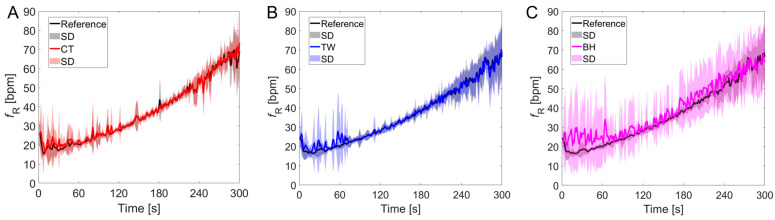
Time course of $f_{R}$ measured with the CT vest (**A**), the TW vest (**B**), and the BH strap (**C**) vs. the reference system during the paced-breathing protocol. Data are expressed as mean ± SD.

**Figure 6:**
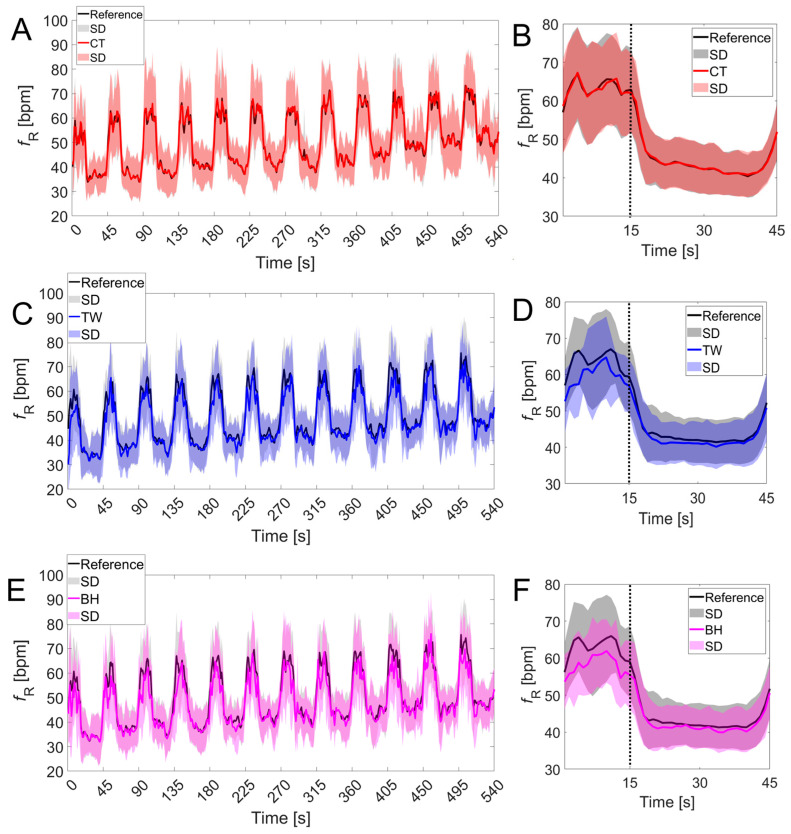
Comparison of the $f_{R}$ time course measured with the commercial devices vs. the reference system during the intermittent test. Data were plotted as a function of time and as a function of the work–rest cycle (15 s of work and 30 s of rest) in the left panels and right panels, respectively. Upper, middle, and lower panels refer to the CT vest (**A**,**B**), the TW vest (**C**,**D**), and the BH strap (**E**,**F**), respectively. The vertical dashed line separates the 15 s of work from the 30 s of rest. Data are expressed as mean ± SD.

Figure 5 and Figure 6 show how the $f_{R}$ time course obtained from the commercial devices generally resembled that of the reference system during the paced-breathing phase and the intermittent test, respectively. However, higher interindividual variability was found for the BH values during paced breathing, as outlined by the higher SD values (Figure 5C). One participant did not perform the paced-breathing task correctly and was not included in the analysis related to that phase. During intermittent exercise, the time course of $f_{R}$ measured with the reference system was best resembled by that of the CT vest, while a mild underestimation of $f_{R}$ was found for TW and BH, especially at $f_{R}$ values above 60 bpm. One participant wearing the TW vest and the BH strap had a technical problem during the intermittent test and missed two repetitions; data from this test were not included in Figure 6C–F but were considered for the other analyses.

### 3.3. MAPE Values across Phases and Window Lengths

**Figure 7:**
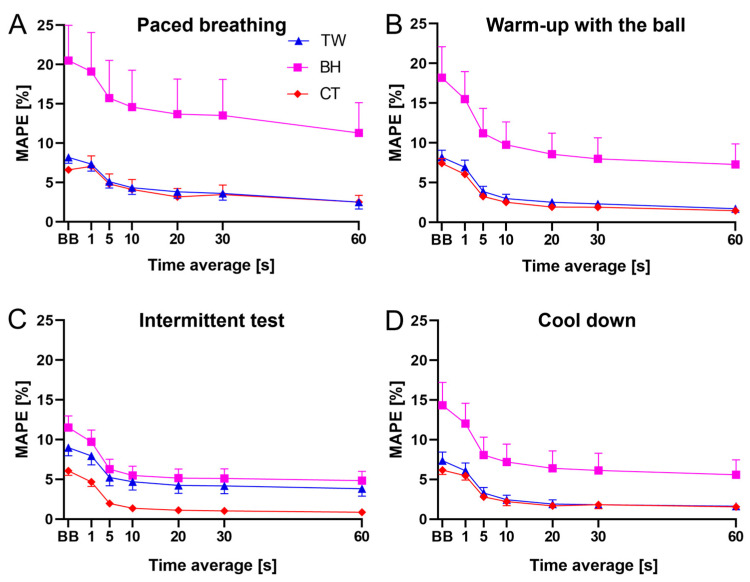
MAPE values for breath-by-breath (BB) data and for different window lengths ranging from 1 s to 60 s during paced breathing (**A**), warm-up with the ball (**B**), intermittent test (**C**), and cool-down (**D**). The CT vest, the TW vest, and the BH strap are represented in red, blue, and magenta, respectively. Data are mean ± SEM.

Figure 7 shows how MAPE values change when varying the window length used to compare the reference system and the device under validation. For all three commercial devices, the highest MAPE values were observed when comparison was made on a breath-by-breath basis, while the lowest MAPE values were observed when comparisons were made based on 60 s windows. Lower MAPE values were found for CT vs. BH in all of the phases of the validation protocol. The performance of the TW vest was, on average, similar to that of the CT vest in all the phases except for the intermittent test, where higher MAPE values were found for the TW vest.

### 3.4. Individual Values of Precision and Accuracy

**Figure 8:**
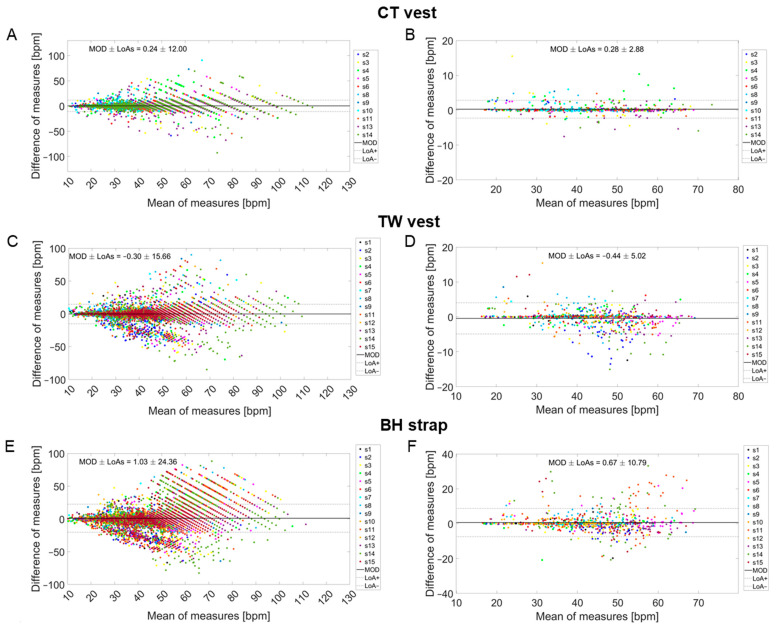
Bland–Altman plots for breath-by-breath data (**left** panels) and data averaged over 30 s (**right** panels) considering the entire validation protocol for the CT vest (**A**,**B**), the TW vest (**C**,**D**), and the BH strap (**E**,**F**). The different colors represent different participants.

Considering the entire validation protocol, both accuracy and precision were better for CT and TW compared to BH, as shown by the higher MOD and LoAs values found for BH (Table 1, Table 2 and Table 3, Figure 8). Interindividual variability in LoAs, MAE, and MAPE values was observed for the three devices, especially for the BH strap (Table 3). One participant did not perform the CT session, while technical problems occurred for three other participants wearing the CT vest, thus preventing the possibility of comparing the CT signal with the reference signal. Hence, the comparison between the CT signal and the reference signal was made for 11 participants, as reported in Table 2. Nevertheless, data loss for CT had no influence on the number of participants considered for TW and BH (i.e., 14 and 15 respectively) as no direct comparison was made between the three commercial devices.

## 4. Discussion

This study assessed the validity of three commercial wearable sensors measuring $f_{R}$ during soccer-specific movements. The requirements of soccer were carefully taken into consideration when designing the study and defining the validation methodology. Soccer is characterized by unorthodox movements, changes of direction, accelerations, decelerations, and torso movements potentially contaminating the respiratory signal with breathing-unrelated artifacts. Furthermore, the intermittent nature and high demand of soccer lead to fast variations in $f_{R}$ that need to be captured appropriately [17]. For this reason, a time domain algorithm was used for breath-by-breath estimation of the $f_{R}$. The accuracy and precision of $f_{R}$ measurement were assessed on a breath-by-breath basis during paced breathing (ranging from 15 to 75 bpm), warm-up with the ball, intermittent shuttle runs, and a low-intensity cool-down. Our findings support the suitability of measuring $f_{R}$ during soccer with commercial wearable sensors. The ComfTech^®^ vest and the Tyme Wear^TM^ vest showed superior performance compared to the Bioharness^TM^ 3.0 strap because the latter generally showed a higher measurement error, especially when the comparison with the reference signal was performed breath by breath.

### 4.1. ComfTech^®^ Vest

The good performance of the CT vest can be appreciated from the results of the different analyses performed. The respiratory waveform recorded from this device shows a good signal-to-noise ratio, as outlined by the relatively small changes in false positives and false negatives when varying the prominence percentage used to extract breath-by-breath $f_{R}$ values. Furthermore, relatively low MAPE values were generally found even at $f_{R}$ levels above 60 bpm. As such, the CT vest showed good performance in monitoring the fast changes in $f_{R}$ observed during intermittent exercise performed at high intensities, as shown in Figure 6. This was evident despite the CT vest having a lower sampling frequency compared to the other two devices (i.e., about 13 Hz vs. 25 Hz). As soccer-specific movements may result in $f_{R}$ values higher than those observed during other exercise modalities at high intensity [17,20,23], the precision and accuracy in detecting high $f_{R}$ values should be prioritized when developing or selecting wearable sensors measuring $f_{R}$ in this context. We are not aware of previous studies validating the CT vest during exercise, but the good performance of this device encourages its assessment in other sporting activities. Indeed, the performance of the CT vest appears to be similar or even superior to that of some other commercial wearable devices tested during scenarios less challenging than soccer (e.g., cycling on an ergometer) [39,40,42].

### 4.2. Tyme Wear^TM^ Vest

Good performance was observed for the TW vest, although the amplitude of the respiratory waveform obtained from this device was relatively low in some instances, especially during high-intensity intermittent shuttle runs. As such, the TW signal is prone to false negatives, especially when the prominence percentage increases, meaning that a progressively increasing number of real breaths may not be detected by the algorithm. This problem is at least partially counteracted when selecting a low prominence percentage (i.e., 2% in this study), which is justified in light of the small number of false positives found. Hence, the signal-to-noise ratio of the TW respiratory waveform is good to measure $f_{R}$ during exercise. However, an underestimation of $f_{R}$ was observed at $f_{R}$ levels above 60 bpm during the high-intensity intermittent test, and MAPE values were higher during this phase of the validation protocol compared to the MAPE values found for CT. Hence, further developments of the TW vest are encouraged to improve even more its suitability in monitoring high-intensity soccer-based activities. The fact that the number of false negatives changes substantially with the prominence percentage used to extract $f_{R}$ from the respiratory waveform suggests that the algorithm considerably impacts $f_{R}$ and the related measurement error. A previous version of the TW device (i.e., a smart shirt) was validated in a study aiming to estimate the ventilatory thresholds from the ventilatory variables recorded with the shirt during a running incremental test [43]. However, no direct validation of the $f_{R}$ measurement was attempted, thus making it difficult to compare our results with those of the previous study.

### 4.3. Bioharness^TM^ 3.0 Strap

The error of measurement of the BH strap was generally higher than that of the CT and TW vests. The BH respiratory waveform is more affected by motion artifacts induced by soccer-based movements compared to the signal of the other two devices. This is reflected in the relatively high values of false positives, especially when using low prominence values (i.e., 1% or 2%). On the other hand, the selection of higher prominence values (10% was chosen in this study) reduces MAPE values and does not increase false negatives excessively. Hence, the signal-to-noise ratio of the BH respiratory waveform is generally good enough to measure $f_{R}$ during exercise, although interindividual differences in signal quality were observed. However, relatively large errors were observed at low $f_{R}$ values (below 25 bpm). Moreover, an underestimation of $f_{R}$ was found at $f_{R}$ values above 60 bpm, especially during high-intensity intermittent exercise. This suggests that the BH device is not an optimal choice when aiming to monitor soccer or other running-based sports with similar demands. The BH strap is the only device among the three tested in this study that has been validated in several exercise studies [33,34,39,40]. The error values reported by previous studies were unsurprisingly generally lower than those observed herein [33,34,39,40], and two main factors largely explain this difference. First, soccer-based activities challenge the respiratory signal more than cyclic activities like walking, running, and cycling. Second, soccer leads to higher $f_{R}$ values, and this contributes to the increase in MAPE values, as shown in Figure 3. Importantly, the lower performance compared to previous studies is more evident when the comparison is made on a breath-by-breath basis [40] rather than based on time windows of several seconds [33,34]. These findings reinforce the proposition that the BH strap is not the most effective device among those tested in this study in monitoring fast changes in $f_{R}$ during soccer activities.

We cannot exclude that the lower performance of the BH device may be attributed to the fact that the strain sensor was integrated into a strap rather than into a vest, although this proposition remains speculative at present. While a strap may be more prone to displacement, no noticeable problems were observed with the BH strap in this regard. On the other hand, a sensor integrated into a vest may be more susceptible to torso movements depending on the specific modalities of integration into the textile. Different sensor characteristics, body locations, and electronics may have contributed to the different performance observed across devices, but it is beyond the scope of this study to identify the specific sources of these differences.

### 4.4. Validation Methodology

Our findings have important implications for the development and validation of sensors and related algorithms to be used in specific sports contexts. We have shown how the measurement error may change substantially depending on the characteristics of the algorithm used (i.e., prominence percentage values), the window length selected to compare the device with the reference system (from breath by breath to 60 s), the $f_{R}$ values (low vs. moderate vs. high), and the specific activities determining different motion artifacts and $f_{R}$ time courses (e.g., paced breathing vs. intermittent exercise). These findings show that the evaluation of the performance of a wearable device is facilitated when a comprehensive validation methodology is employed. We have also used a breath-by-breath method of comparison that distinguishes between false positives (i.e., breaths detected but not real) and false negatives (real breaths not detected by the device under validation), which is valuable for refining the algorithm to be used in the context of interest. A specific example is how we have chosen the prominence percentage for the three commercial devices based on a trade-off between the reduction in false negatives and the increase in false positives. The importance of validating devices on a breath-by-breath basis cannot be overlooked considering the demands of soccer and the rapid fluctuations in $f_{R}$. Hence, the development of wearable devices and related algorithms should consider the specific needs of soccer or, in general, the sports discipline of interest.

## 5. Conclusions

This study shows the suitability of monitoring $f_{R}$ during soccer-specific movements with some commercially available devices integrating strain sensors. The CT vest generally showed a lower measurement error than the other two devices, especially during high-intensity intermittent exercise. Good performance was also observed for the TW vest, while the BH strap generally showed a higher measurement error. Furthermore, we developed an algorithm for $f_{R}$ estimation that can be embedded in a stand-alone device, thus enabling online data processing. Our findings outline the importance of developing sensors and algorithms for the specific needs of soccer monitoring. Considering the good wearability of the devices tested, our findings pave the way for breathing monitoring during soccer and other running-based team sports.

## Figures and Tables

**Table 1:** TW vest; MOD ± LoAs, MAE, and MAPE values for single participants. Overall values are reported in bold.

	Breath-by-Breath Analysis			Analysis Based on 30 s Windows		
Soccer Player	MOD ± LoAs [bpm]	MAE [bpm]	MAPE [%]	MOD ± LoAs [bpm]	MAE [bpm]	MAPE [%]
1	−0.11 ± 8.14	1.88	5.16	−0.24 ± 3.82	0.56	1.44
2	−1.75 ± 19.32	5.67	11.81	−2.62 ± 7.75	3.17	6.39
3	−0.43 ± 17.91	4.28	9.36	−0.53 ± 3.82	1.25	2.98
4	0.19 ± 13.42	3.33	7.85	0.10 ± 3.03	1.01	2.76
5	−0.52 ± 13.12	3.37	6.14	−0.69 ± 2.89	0.86	1.57
6	0.18 ± 11.07	2.68	7.13	0.07 ± 2.79	0.66	1.66
7	0.08 ± 9.83	2.55	6.05	0.01 ± 2.26	0.55	1.42
8	−0.12 ± 18.25	4.66	11.95	0.05 ± 5.73	2.11	5.58
9	0.08 ± 10.37	2.82	6.37	0.07 ± 2.63	0.51	1.70
11	−0.44 ± 15.89	4.29	8.40	−0.69 ± 3.78	1.30	3.07
12	0.11 ± 11.10	2.78	7.51	0.13 ± 5.31	1.21	3.68
13	−0.43 ± 19.34	5.47	10.89	−0.53 ± 4.83	1.74	3.28
14	−0.94 ± 24.67	6.68	13.26	−1.28 ± 8.59	3.01	6.19
15	0.03 ± 15.48	4.00	9.16	0.01 ± 5.56	1.35	4.09
**Overall**	**−0.30 ± 15.66**	**3.89**	**8.65**	**−0.44 ± 5.02**	**1.38**	**3.27**

**Table 2:** CT vest; MOD ± LoAs, MAE, and MAPE values for single participants. Overall values are reported in bold.

	Breath-by-Breath Analysis			Analysis Based on 30 s Windows		
Soccer Player	MOD ± LoAs [bpm]	MAE [bpm]	MAPE [%]	MOD ± LoAs [bpm]	MAE [bpm]	MAPE [%]
2	0.20 ± 7.37	1.94	4.81	0.27 ± 1.55	0.36	1.16
3	0.08 ± 12.97	3.75	7.32	0.28 ± 4.90	1.02	3.72
4	1.15 ± 17.04	4.49	10.17	1.10 ± 4.30	1.35	3.23
5	0.23 ± 9.66	3.22	6.18	0.24 ± 1.40	0.36	1.07
6	0.21 ± 8.62	2.39	6.35	0.10 ± 1.27	0.29	0.89
8	0.28 ± 8.03	2.42	6.36	0.43 ± 2.26	0.64	2.24
9	0.16 ± 7.44	2.18	5.28	0.23 ± 1.63	0.31	1.01
10	0.37 ± 10.02	2.83	8.06	0.53 ± 2.50	0.59	1.79
11	0.22 ± 10.43	3.16	6.10	0.20 ± 1.75	0.47	1.01
13	−0.31 ± 15.39	4.12	8.17	−0.37 ± 3.39	1.00	2.15
14	0.28 ± 16.51	4.36	8.54	0.16 ± 3.16	0.92	2.12
**Overall**	**0.24 ± 12.00**	**3.17**	**7.03**	**0.28 ± 2.88**	**0.66**	**1.85**

**Table 3:** BH strap; MOD ± LoAs, MAE, and MAPE values for single participants. Overall values are reported in bold.

	Breath-by-Breath Analysis			Analysis Based on 30 s Windows		
Soccer Player	MOD ± LoAs [bpm]	MAE [bpm]	MAPE [%]	MOD ± LoAs [bpm]	MAE [bpm]	MAPE [%]
1	−0.05 ± 12.11	2.64	6.98	−0.21 ± 5.91	0.90	2.02
2	−1.93 ± 18.16	5.23	10.47	−2.44 ± 5.14	2.49	5.05
3	0.25 ± 28.54	8.49	17.57	−0.12 ± 8.28	3.01	7.42
4	0.13 ± 12.11	3.53	8.07	−0.26 ± 6.00	1.25	3.07
5	2.38 ± 25.97	8.19	15.74	2.15 ± 10.12	3.21	7.62
6	0.17 ± 17.02	4.36	10.78	0.05 ± 3.51	1.25	3.68
7	0.19 ± 8.14	2.53	6.05	0.12 ± 1.32	0.32	0.95
8	2.03 ± 23.37	6.76	16.81	2.26 ± 6.08	2.75	7.70
9	0.16 ± 13.04	3.45	7.44	0.13 ± 3.71	0.87	1.94
10	0.31 ± 12.68	3.15	8.83	0.35 ± 2.97	0.89	2.86
11	7.84 ± 43.16	16.98	34.30	5.53 ± 22.44	10.04	21.5
12	0.40 ± 13.47	3.81	9.69	0.45 ± 5.07	1.30	4.01
13	−0.62 ± 20.86	6.31	12.43	−1.24 ± 4.84	2.12	4.43
14	2.91 ± 40.73	13.62	30.78	2.93 ± 22.49	9.28	23.89
15	0.92 ± 32.01	9.77	23.03	0.36 ± 13.94	4.54	13.4
**Overall**	**1.03 ± 24.36**	**6.59**	**14.60**	**0.67 ± 10.79**	**2.95**	**7.30**

## Data Availability

The data presented in this study are available upon request from the corresponding author. The data are not publicly available due to privacy reasons. The MATLAB^®^ functions are available upon request from the corresponding author.
